# Nutrition, Lipoproteins and Cardiovascular Diseases

**DOI:** 10.3390/nu16152530

**Published:** 2024-08-02

**Authors:** Abdelouahed Khalil

**Affiliations:** Geriatrics Unit, Department of Medicine, Faculty of Medicine and Biological Sciences, University of Sherbrooke, Sherbrooke, QC J1H 4N4, Canada; abdelouahed.khalil@usherbrooke.ca

Cardiovascular diseases (CVDs) represent the leading cause of mortality worldwide, despite the significant advancements that have been made in terms of primary and secondary prevention strategies over the past decades. Pharmacological options are often the treatments that are initially presented; however, it is relevant to note that non-pharmacological interventions, such as physical activity and diet, are associated with a substantial reduction in cardiovascular events. Therefore, more attention should be paid to the study of strategies for the primary prevention of CVDs to allow us to select the most effective interventions, whether these are through the use of functional foods or nutraceuticals, to provide protection against these diseases.

Reducing a patient’s levels of low-density lipoproteins (LDLs) has been the cornerstone of CVD prevention since the discovery of statins in the 1980s. However, statin treatments have certain limitations which are related to their side effects and the patient populations that are eligible to use them, particularly the elderly population [1]. Moreover, they are most often used in secondary prevention. In contrast, the role of high-density lipoproteins (HDLs) in cardiovascular protection is gaining increasing interest. A low level of HDLs has now been established as a powerful risk factor for myocardial infarction, independently of LDL levels. However, studies of pharmacological interventions aimed at increasing plasma levels of HDLs have yielded conflicting or disappointing results. Conversely, clinical studies have shown that improving the functionality of HDLs is much more important than increasing their concentration in reducing the risk of cardiovascular events [2,3]. This Special Issue is dedicated to highlighting the beneficial effects of certain nutrients, notably polyphenols, on cardiovascular health and addresses the link between LDL and HDL metabolism and the development of the atherosclerosis process. It comprises a total of eight scientific articles from eminent researchers in the field of atherosclerosis and associated diseases.

In this context, Morvaridzadeh et al.‘s paper [4] presents a comprehensive review of the metabolism of HDLs and their function in relation to their anti-atherosclerotic properties. Their literature review focuses on conducting a structural and biochemical analysis of HDLs and the parameters that regulate HDLs’ functionality. Special attention is given to the ability of HDLs to facilitate reverse cholesterol transport (RCT). RCT represents a unique pathway for the removal of excess cholesterol [5] and confers anti-atherosclerotic properties to HDLs [6]. On the one hand, this function of HDLs involves the activity of enzymes associated with HDLs, notably LCAT (lecithin-cholesterol acytransferase) and CETP (Cholesteryl ester transfer protein), which are involved in the process of HDL maturation and lipid enrichment. On the other hand, it relies on the interaction of apo-A1, a specific protein found within HDLs, with cholesterol membrane transporters and receptors (ABCA1, ABCG, SR-BI). Finally, Morvaridazdeh et al.’s paper [4] focuses on the changes that occur with age which can impact the development of atherosclerosis, and more specifically, how these changes can affect the functionality of HDLs. The paper concludes with a discussion of the effects of diet on cardiovascular protection, placing increased emphasis on the effect of certain nutrients on HDL functionality and on the activities of enzymes associated with HDL (CETP and LCAT). 

Teresa Padro et al.’s paper [7] addresses, through a longitudinal crossover clinical study, how dietary an omega-3 fatty acids-rich diet modulates the HDL lipidome compared to a phytosterols-rich diet. Their results showed a significant change in the levels of lipidomic HDL following a 28-day intervention with an omega-3-rich diet (docosahexaenoicacid: DHA an eicosapentaenoic acid: EPA). The importance of these results stems from the fact that the functionality of HDL, particularly its cholesterol efflux capacity, is highly dependent on the overall HDL lipidome [8]. Moreover, among the 333 species of lipids that form HDLs, 209 are associated with the size of the HDLs. Although the number of metabolites (263) identified by Padro et al. was significantly lower than those established by others (333), their results showed that four weeks of intervention with an omega-3-rich diet induced changes in the levels of 35 HDL lipid metabolites, accounting for approximately 13.5% of the lipid metabolites that form HDLs. The results of the study by Padro et al. also demonstrate sex differences in the HDL lipidome both at baseline and after a dietary intervention [7]. However, further studies are needed to confirm the reliability of these results in a larger population and to investigate the impact of sex hormones on HDLs’ lipid compositions.

Zimmer et al.‘s [9] study focused on the effect of a diet rich in extra virgin olive oil (EVOO) on the inflammatory profile of monocytes in elderly patients at high risk for cardiovascular events, particularly those with hypercholesterolemia or those who had previously suffered a heart attack. They investigated the activation of monocytes, a key step in the process of atherosclerosis, and the recruitment of these monocytes into the intima where they differentiate into macrophages within the plaque. This differentiation also exacerbates the inflammatory process that occurs during a cardiovascular event. Zimmer et al.’s [9] results show that dyslipidemia patients have monocytes that have a higher inflammatory status than those in a control group (healthy participants) or post-heart attack patients. Interestingly, the results of this study show that 6 months of supplementation with EVOO has no effect on the phenotype of the monocytes or on cytokine production.

Epidemiological studies have highlighted the cardioprotective effect of EVOO and have largely attributed this effect to its richness in polyphenols. Metabolic syndrome represents a cluster of several cardiovascular risk factors, which includes a significant waist circumference, high blood pressure, insulin resistance, and dyslipidemia. Polyphenols are increasingly targeted as nutrients that are capable of regulating these CVD risk factors, [10,11]. Pomegranate, which is highly rich in polyphenols, including ellagitannins, ellagic acid, and other flavonoids (quercetin, kaempferol, and luteolin glycosides), has been suggested to present cardioprotective effects [12]. Alami et al.’s review [13] discusses the pharmacokinetic properties and the catabolism, absorption, biodistribution, and clearance of Ellagitannins. The anti-hyperlipidemic and normoglycemic molecular mechanisms of the phytochemical compounds that are present in pomegranates are also presented. Finally, Alami et al.’s paper summarizes the principal animal and clinical studies that have been carried out to discern the effect of pomegranate consumption on regulating hyperlipidemia, preventing diabetes, obesity, and in protecting against CVDs.

In addition to HDLs, whose atheroprotective roles have been widely demonstrated, a high level of LDLs is associated with an increased risk of cardiovascular events, hence their identification as pro-atherogenic lipoprotein classes. However, lipoprotein (a) (Lp(a)), a form of LDL, is even more atherogenic than LDL itself. The plasma level of Lp(a) is genetically defined [14], and a high level of Lp(a) promotes inflammation and coagulation, significantly increasing the risk of myocardial infarction, aortic stenosis, stroke, and peripheral arterial disease [15,16]. The study by Aljawini et al. [17] focused on measuring the level of Lp(a) in a less explored population, namely Saudi women, to determine both the effect of age and menopause on the levels of Lp(a) and predictors of elevated Lp(a) in this population. Their results show that age significantly predicts high Lp(a) levels in women. Besides this study focusing solely on women and the effect of menopause, the obtained results provide another contribution to understanding the effect of age as a risk factor for cardiovascular diseases.

A healthy diet is effective both in primary prevention of CVDs and in secondary prevention. In relation to this, Czinege et al. [18] conducted a prospective study on the impact of nutritional status on the incidence risk of cardiovascular complications among high-risk cardiovascular patients (post-myocardial infarction patients). Their findings have established a connection between the nutritional status, the inflammatory state, and the recurrence of a cardiovascular event. Malnourished patients are at a higher risk of developing cardiovascular complications after their first myocardial infarction. These results emphasize the importance of nutrition as a major determinant both in protecting against CVDs and in facilitating recovery after a patient’s first myocardial infarction. 

Continuing along the same vein, Seeba et al. [19] investigated how a vegan diet compared to an omnivorous diet may be protective against CVDs. Their results showed that although the vegan diet was associated with a reduction in cholesterol levels, no significant differences were observed in the activity of lipoprotein lipase (LPL). LPL is implicated in the regulation of lipid homeostasis, primarily by mediating the intravascular lipolysis of triglyceride. 

Finally, the paper by Jacques and Bkaily [20] focuses on the beneficial effect of taurine in the prevention of cardiac hypertrophy. Their hypothesis suggests that taurine is a potent antioxidant that can protect against dysfunction of cardiac endothelium. This effect would involve the regulation of intracellular calcium levels and the prevention of an overload of reactive oxygen species. To verify their hypothesis, Jacques and Bkaily used isolated endothelial cells from human heart ventricles in combination with confocal microscopy to both measure changes in the volumes of different cellular compartments and to quantify the levels of Ca^2+^ and ROS using specific markers. The confirmation of taurine’s protective effect against cardiac hypertrophy is very promising for the treatment of Duchenne muscular dystrophy.

In conclusion, all eight these articles provide an original contribution to ongoing attempts to elucidate the role of certain nutrients in protecting against cardiovascular diseases. The review articles presented in this Special Issue also summarize the main advances that have been made in this field.

## Data Availability

Data are contained within the article.
